# An iterative workflow for creating biomedical visualizations using Inkscape and D3.js

**DOI:** 10.1186/1471-2105-16-S15-P10

**Published:** 2015-10-23

**Authors:** William A Mattingly, Robert R Kelley, Julia H Chariker, Tim Weimken, Julio Ramirez

**Affiliations:** 1 Division of Infectious Diseases, University of Louisville, Louisville, KY 40202, USA; 2 Department of Psychological and Brain Sciences, University of Louisville, Louisville, KY 40292, USA; 3 KBRIN Bioinformatics Core, 522 East Gray Street, University of Louisville, Louisville, KY 40202, USA

## Background

**Figure 1:**
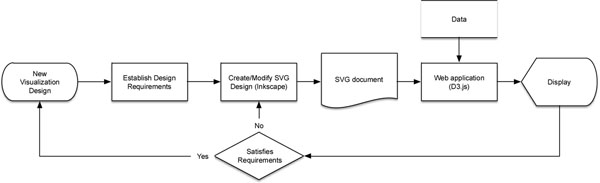
Workflow diagram.

Many biological disciplines use data visualization alongside computational methods to explore large-scale biomedical data. Visualization often provides insight into patterns in the data that are not available in the numerical data and statistics[1]. The development of new visualization tools requires the use of sophisticated software and programming skills. Commercial standalone software like Tableau[2] create multiple types of common visualizations and have the ability to customize certain features. There are also freely available software libraries like D3.js[3] that can be used to make interactive web applications based on static or dynamic data. Nevertheless, modern data visualization is highly sophisticated, and creating customized visualizations to interact with a specific dataset can be challenging for a variety of reasons. Specifically with D3.js, which builds a scalable vector graphic (SVG) programmatically, generating the visualization is a process of trial and error. The programmer generates SVG markup manually and then views it with a browser and does this iteratively until the final graphic is realized. We present an iterative workflow shown in Figure 1 that simplifies the creation of SVG images using freely available software. We demonstrate this workflow in constructing an interactive dashboard to track clinical trial enrollment.

## Materials and methods

**Figure 2:**
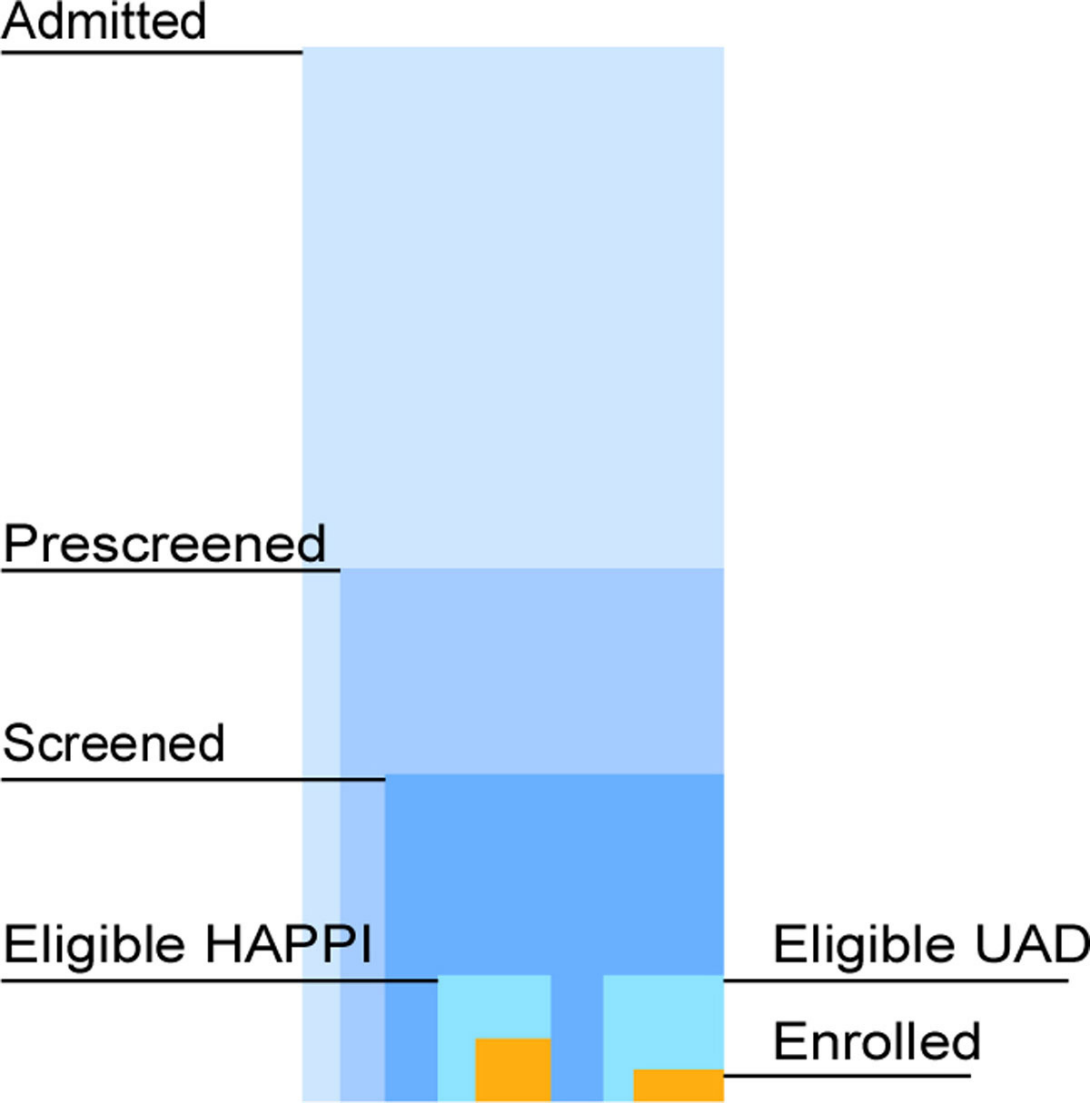
Layered bar graph created in Inkscape for use in clinical dashboard.

Using the Scalable Vector Graphics (SVG) language[4] and the open source SVG authoring tool Inkscape[5], we created and tested several prototypes for visualizing clinical trial enrollment across nine adult hospitals in Jefferson County. The information required by a clinical trial manager included the number of total enrollments per hospital and other contextual data related to the number of enrollments such as the numbers admitted, screened and eligible for the two trials, UAD and HAPPI. A layered bar graph design, shown in Figure 2, provided an efficient method for displaying the necessary information within the appropriate context and in the smallest space. After creating the mockup of the system in Inkscape, it is saved as an SVG document and imported into a live website. Inkscape assigns variable names to each primitive object at the time of creation. The D3.js library can then be used to access the properties of these objects and manipulate them according to the data.

## Results

The enrollment dashboard prototype was created over the course of 1 week. Many hours were saved on the development of each feature by allowing the design of SVG prototypes without needing to learn the language-specific layout syntax.

## Conclusions

SVG prototypes developed in Inkscape can be adapted for use with advanced visualization libraries like D3.js to form an iterative workflow for creating customized visualizations and dashboards. While manipulating interactive SVG still requires knowledge of JavaScript, our approach significantly reduces the development time.

## References

[B1] Anscombe FJ Graphs in Statistical Analysis American Statistician 1976 27 1 17 21

[B2] Bostock M D3.js – Data-Driven Documents [http://d3js.org] Accessed 22 Feb 2015 10.1109/TVCG.2011.185 22034350

[B3] Tableau Software Business Intelligence and Analytics [http://www.tableau.com] Accessed 22 Feb 2015

[B4] Inkscape [https://inkscape.org] Accessed 22 Feb 2015

[B5] W3C SVG Working Group [http://www.w3.org/Graphics/SVG] Accessed 22 Feb 2015

